# p53 as a potential predictive factor of response to chemotherapy: feasibility of p53 assessment using a functional test in yeast from trucut biopsies in breast cancer patients

**DOI:** 10.1038/sj.bjc.6600105

**Published:** 2002-03-04

**Authors:** H Bonnefoi, A Ducraux, S Movarekhi, M F Pelte, S Bongard, E Lurati, R Iggo

**Affiliations:** Département de Gynécologie Obstétrique, Hôpitaux Universitaires de Genève, Boulevard de la Cluse 32, CH-1211 Genève 14-Switzerland; Département de Pathologie-CMU, Hôpitaux Universitaires de Genève, Rue Michel-Servet 1, CH-1211 Genève 14-Switzerland; Swiss Institute for Experimental Cancer Research (ISREC), Chemin des Boveresses 155, CH-1066 Epalinges-Switzerland

**Keywords:** p53, neoadjuvant chemotherapy, yeast assay, breast cancer

## Abstract

Assessment of the predictive value of p53 requires the testing of large numbers of samples from patients enrolled in prospective phase III clinical trials. The goal of this study was to determine whether p53 status can be determined by p53 yeast functional assay using the limiting amounts of material that can typically be obtained in prospective phase III trials (particularly when chemotherapy is given before surgery). All patients presenting with a clinically palpable tumour which could be considered large enough to perform a trucut biopsy (⩾2 cm breast tumour) were eligible for this study. Two trucut biopsies and one incisional biopsy were performed on the surgical specimens (mastectomy or tumourectomy). Samples were snap frozen and cryostat sections were taken for histology and p53 testing. Thirty patients were included. Three samples out of 90 failed to give any p53 PCR products, probably because these samples contained almost entirely fibrous tissue. Of the 87 samples that could be tested, the incisional and trucut biopsies results were fully concordant in every case. p53 could be defined in 97% of patients by double trucut biopsy. Eight out of 30 tumours tested were mutant for p53 (27%). p53 status can be reliably determined by yeast assay from single frozen sections of trucut biopsies. Histological examination before p53 testing is essential to exclude cases where the p53 result may reflect only the status of the normal cells in the biopsy.

*British Journal of Cancer* (2002) **86**, 750–755. DOI: 10.1038/sj/bjc/6600105 www.bjcancer.com

© 2002 Cancer Research UK

## 

Experimental and clinical studies have shown that anticancer agents strongly induce apoptosis (Hickman, 1992; Ellis *et al*, 1997). p53 is a key regulatory gene in the apoptotic pathway, and experimental data have shown that tumours containing wild type p53 respond better to anthracyclines than p53-mutant tumours (Lowe *et al*, 1993, 1994; Gudas *et al*, 1996). In contrast, most *in vitro* and *in vivo* studies have shown that p53 status does not affect the response to taxanes (Woods *et al*, 1995; Brown, 1996; Jordan *et al*, 1996; Perego *et al*, 1996; Wahl *et al*, 1996; Lanni *et al*, 1997; O'Connor *et al*, 1997; Fan *et al*, 1998).

Taken together, these experimental results suggest that p53 status may help to predict the response to chemotherapy with anthracyclines or taxanes in clinical practice. Specifically, one can make the hypothesis that p53 mutant tumours will be anthracycline-resistant but taxane-sensitive. Several clinical studies addressing this question have failed to demonstrate a predictive value of p53. Two clinical studies where the p53 DNA sequence was assessed suggested that p53 status may be important. The first study found that p53 mutant tumours were resistant to anthracyclines (Aas *et al*, 1996) and the second found that p53 mutant tumours were sensitive to taxanes (Kandioler-Eckersberger *et al*, 2000). However the number of patients analysed in these studies was too small for definitive conclusions to be drawn.

In order to address this question definitively in breast cancer we have initiated a large phase III clinical trial (EORTC 10994/BIG 00-01 study). Testing p53 in the context of a phase III clinical trial poses major technical and logistical problems which deserve comment. First, correlation of a single biological variable with response to different treatments, the minimum design to show a predictive effect, requires four times as many patients as a standard two treatment comparison (Peterson and George, 1993). Making reasonable assumptions about the likely prognostic and treatment effects of p53 status in patients with locally advanced breast cancer, the number of patients required to answer the p53/taxane question in 3 years is about 1400. Second, although widely available, immunohistochemistry (IHC) is not the best method to assess p53 status. The risk of false-positive and false-negative results is higher with IHC than sequencing (Fisher *et al*, 1994; Sjogren *et al*, 1996; Duddy *et al*, 2000). In particular, IHC fails to detect mutants which encode unstable proteins (nonsense mutations, splicing mutations), which have been found in up to 47% of p53 mutations in breast tumours (Chappuis *et al*, 1999). Simpler DNA structure-based techniques, particularly denaturing gradient gel electrophoresis, are sensitive but require sequencing to show that the change is not a polymorphism or silent mutation. Genomic p53 sequencing is generally considered to be the gold standard against which new p53 assessment methods should be compared, but complete detection of mutations in formalin fixed tissue requires microdissection of tumour samples, a time-consuming procedure when hundreds of samples must be tested (Hartmann *et al*, 1997). We have pioneered the use of an RNA-based technique that detects functionally important p53 mutations (Flaman *et al*, 1995). We amplify p53 cDNA by RT–PCR and then express the cloned DNA in yeast. If the encoded p53 protein is wild type it activates transcription of a reporter gene. The advantage of this technique is that it tests a large number of individual clones, so it can detect mutant p53 mRNA in clinical samples that contain a substantial amount of normal tissue. It is also possible to test the large number of samples required to answer clinical questions, such as the role of p53 in taxane response. Although nonsense-mediated RNA decay renders detection of chain-terminating mutations more difficult with an RNA-based technique, we have shown that the yeast assay is particularly good at detecting this type of mutation (Chappuis *et al*, 1999). Third, collection of samples in a manner that preserves the integrity of RNA is a major problem in a multicentre clinical trial. We have previously shown that p53 status can be accurately determined from frozen sections of incisional biopsies from breast tumours (Chappuis *et al*, 1999). Patients presenting with large operable or locally-advanced/inflammatory breast cancers will be eligible to participate in the EORTC 10994/BIG 00-01 study. Current practice in this group of patients is to use trucut biopsies rather than incisional biopsies. The purpose of the present study was to determine whether it is feasible to test the p53 status of breast tumours by yeast assay in conditions resembling those pertaining in this EORTC clinical trial.

## MATERIALS AND METHODS

Permission for the study was granted by the Geneva University Hospitals ethics committee. All patients gave informed consent to participate in the study. Mastectomy or tumourectomy specimens in a group of 30 patients were taken from the operating theatre to the pathology department with the minimum delay and three biopsies were taken (two trucut biopsies with a 14G needle and one incisional biopsy). The study was performed on surgically resected mastectomy or tumourectomy specimens because ethical concerns preclude multiple sampling of tumours for research purposes in living subjects. Biopsies were embedded in OCT and frozen in 2-methylbutane immediately. Samples for routine diagnosis were taken in parallel and fixed in formalin. Histological assessment of all samples was performed by the same pathologist (MF Pelte). The yeast assay was performed on 100 μm frozen sections essentially as described by Chappuis *et al* (1999). mRNA was purified using oligo-dT Dynabeads (Dynal). Total RNA was purified with Trizol (Life Technologies). Pfu turbo polymerase (Stratagene) was used instead of Pfu polymerase. To rule out a decrease in fidelity of this enzyme induced by the proprietary modification, we performed polymerase fidelity assays (Flaman *et al*, 1994). Consistent with the manufacturer's claims, we could detect no difference in the fidelity of Pfu and Pfu turbo (data not shown). We therefore used Pfu turbo for the present study. The greater processivity meant that a 1 kb p53 cDNA fragment could be amplified as efficiently as the smaller fragments used for the split version of the yeast assay (Waridel *et al*, 1997). We therefore used only the conventional form of the assay, in which a 1 kb p53 PCR product is gap repaired into the pRDI-22 yeast expression vector (Waridel *et al*, 1997).

Sequencing was performed on a minimum of four plasmids rescued from different colonies for each p53 mutant tumour. Sequencing primers were either IF12 and IR13 (Chappuis *et al*, 1999) or IF216 and IR217 (Table 1

**Table 1:**
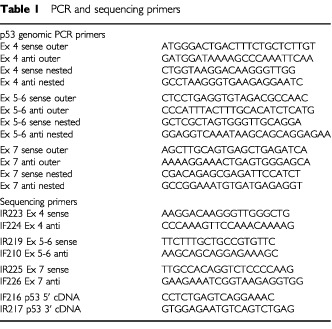
PCR and sequencing primers

), which lie at the beginning and end of the p53 open reading frame and permit sequencing of the entire open reading frame in a single run on a Licor 4200L sequencer. Each plasmid was sequenced on only one strand, but each mutation was visualised on both strands.

To test sample 17B by microdissection, a single frozen section was dissected with a Leica laser microdissector. Genomic DNA was extracted by proteinase K digestion, amplified by nested PCR and exons 4–7 were sequenced directly (see Table 1 for primers).

Quantitative PCR for GAPDH was performed on a PE5700 PCR machine using primers gaaggtgaaggtcggagtc, gaagatggtgatgggatttc, and Taqman probe caagcttcccgttctcagcc. cDNA was synthesised with Superscript reverse transcriptase (Life Technologies) as for p53 (Chappuis *et al*, 1999) except that hexamers were used, and amplified using a Taqman PCR master mix (Perkin-Elmer).

## RESULTS

### Definition of the cut-off for assignment of p53 status

**Figure 1:**
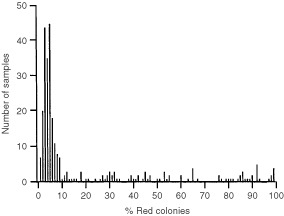
Histogram showing the distribution of the percentage of red for yeast assays on frozen sections of breast tumours. The mean background with wild type samples is 5% red colonies (see text).

One critical parameter in the test is the background number of red colonies. This determines the maximum permissible contamination of tumour samples with genetically normal cells. Histological examination revealed that most samples contained little or no normal breast tissue, but all contained significant numbers of genetically normal cells, principally fibroblasts, inflammatory cells and endothelial cells. Samples frequently contained large fibrotic acellular regions, with tumour cells present in small islands. Precise estimation of the number or fraction of tumour cells is extremely difficult in such heterogeneous material. The observed distribution of red colonies was used to infer the real background with clinical samples. The causes of variation in the background include factors like RNA polymerase errors, splicing artefacts, reverse transcriptase errors, Pfu polymerase errors, variation in the amount of input RNA, gap repair cloning artefacts, recombination at the reporter locus and yeast genetic suppressors of *leu2-3,112*. If we assume that these occur essentially at random, we should see two superimposed distributions in the results: a normally distributed population derived from wild type samples, superimposed upon a flatter distribution derived from p53 mutant samples. The latter can take any value above background, depending on the amount of normal tissue in the sample. This is exactly what is observed. Figure 1

shows the distribution of all results from this study combined with those of Chappuis *et al* (1999), which was also performed on frozen sections of breast tumours. Since we know from sequencing of plasmids rescued from red colonies that samples with ⩾20% red colonies contain clonal mutations, we can disregard these samples and calculate the mean and standard deviation of the remaining samples to obtain an upper limit for the background. The mean is 5%, with a standard deviation of 3%. This value for the standard deviation probably slightly overestimates the true variation because the wild type and mutant distributions overlap.

### p53 status determined by yeast assay

Eight out of 30 tumours tested were mutant for p53 (27%, Table 2

**Table 2:**
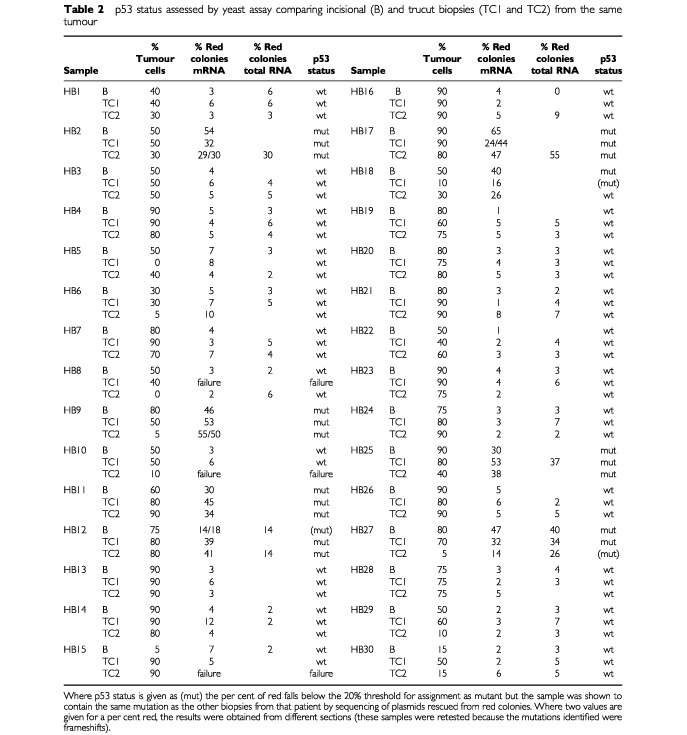
p53 status assessed by yeast assay comparing incisional (B) and trucut biopsies (TC1 and TC2) from the same tumour

). Three samples failed to give any p53 PCR products. Histological examination revealed that these samples contained almost entirely fibrous tissue with very few cells. Of the samples that could be tested, the incisional and core biopsy results were fully concordant in every case (<20% or ⩾20% red colonies) except for HB12, HB18 and HB27, where the percentage of red colonies was between 10 and 19%. Sequencing of plasmids rescued from red colonies revealed that the 187fs mutation was present in the red colonies from all three HB12 samples (B, TC1, TC2), that the 266E mutation was present in all three HB18 samples (B, TC1, TC2) and that the 278S mutation was present in all three HB27 samples (B, TC1, TC2). The low percentage of red colonies in the discordant samples was thus due to a low tumour cell content, rather than a high background intrinsic to the assay. Plasmids were rescued from red colonies in every other case where the mutant content was ⩾20%. The same mutation was found in every sample from a given tumour, again confirming that the use of frozen sections of biopsies is perfectly compatible with assignment of p53 status by yeast functional assay.

The analysis above was performed with mRNA purified on paramagnetic beads. Since mRNA purification is extremely sensitive to RNA quality, we retested 54 samples for which material was available using total RNA (Table 2). The yield of RNA measured by a quantitative PCR assay for GAPDH was higher with total than messenger RNA (median 27-fold, interquartile range 7.6- to 101-fold), suggesting that RNA degradation may indeed be a problem in the biopsies. Except for HB12 TC2 and HB27 TC2, the assignment of p53 status was identical using mRNA and total RNA, and the background was the same. Both HB12 and HB27 contain mutant p53, but in one case the percentage red colonies fell below the 20% cut-off and in the other it rose above it. This is most likely explained by changes in the mutant content at different levels in the biopsy. Other groups have also obtained satisfactory results with the yeast assay using total RNA extracted from tumours (Tada *et al*, 1996; Kashiwazaki *et al*, 1997; Obata *et al*, 2000).

### Mutation type

The base changes are given in Table 3

**Table 3:**
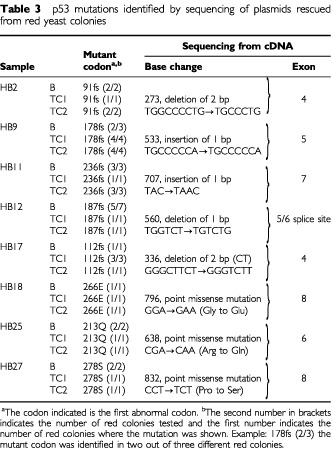
p53 mutations identified by sequencing of plasmids rescued from red yeast colonies

**Figure 2:**
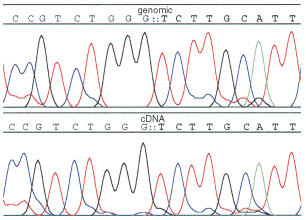
Genomic and cDNA sequence showing the mutation in sample 17B.

. Only three of the eight p53 mutations identified were classic missense mutations. The remainder were frameshift mutations. This is an unusually high proportion of frameshifts, compared to the data in the literature (IARC database, Hernandez-Boussard *et al*, 1999). This high proportion may be due to chance in this small series or to the fact, as we previously noted, that the yeast assay is particularly good at detecting non-missense mutations (Chappuis *et al*, 1999). Several arguments point to the mutations being real. First, the same mutation was present in three different samples (the incisional biopsy and two trucut biopsies). Second, we retested one biopsy from four of the five patients with frameshift mutations and confirmed the result (HB2, HB9, HB12, HB17; in HB11 there was insufficient material for retesting) (Table 2). The exact percentage of red was not the same on retesting because the mutant content varies at different levels in the biopsy. In HB12 TC2 the percentage of red was below the 20% cut-off, but the same mutation was identified by sequencing of plasmids rescued from red yeast colonies on both occasions. Third as a direct test of whether we could confirm the presence of the mutation in HB17, we directly sequenced DNA extracted from a laser microdissected section. This showed that the same mutation was present in the genomic DNA as in the cDNA (Figure 2

). There was insufficient material to test the other samples containing frameshift mutations by microdissection. Taken together, these results make it unlikely that the frameshift mutations were caused by some hidden artefact in the yeast assay.

## DISCUSSION

The main conclusion from this study is that it is possible to test the p53 status of trucut biopsies of breast tumours using a functional assay in yeast. Ninety samples were taken and the yeast assay was successfully performed on 87 of them. In three patients the functional assay failed completely with one of the trucut biopsies, but was successful with the other. Complete failure, meaning we were unable to amplify an RT–PCR product, is most likely due to a combination of RNA degradation and low cellularity of the sample.

The commonest problem was not failure to obtain RNA but a low tumour cell content. It clearly makes no sense to assign a p53 status to samples containing only very small numbers of tumour cells, because of the very substantial risk that the result reflects only the status of the normal cells in the biopsy. For this reason it is essential that a trained pathologist examines samples histologically and excludes those samples with less than a minimum threshold amount of tumour cells. The choice of this threshold is arbitrary, but since we use a 20% red colony cut-off for assignment of p53 status in the yeast assay (see below), it would be reasonable to exclude samples containing less than 20% tumour cells on histological examination. In one case (HB9 TC2), the percentage of red colonies was much higher than the tumour cell content (50 *vs* 5%). Finding a somewhat higher percentage of red colonies than percentage of tumour cell content is normal given the higher rate of transcription in metabolically active tumour cells than normal cells, but a 10-fold difference is unusual. The most likely explanation in HB9 TC2 is that the tumour cell content was higher in the section tested by yeast assay than in the section processed for histology.

The distribution of values for ostensibly wild type samples was used to estimate the background in the assay. The true background should be slightly lower, because some putative wild type samples may have contained low numbers of p53 mutant tumour cells. By selecting a cut-off of 20% red colonies for the prospective clinical trial mentioned in the introduction (EORTC 10994/BIG 00-01 study), we expect to correctly identify all tumours containing wild type p53 (high specificity). A small number of p53 mutant samples with low tumour content may be misclassified (low sensitivity), but there is a good chance, as shown with HB9, HB18 and HB27, that in these cases the second biopsy will contain more tumour cells and give a positive result.

The p53 status assigned (wild type or mutated) was identical whether we used material from trucut biopsies or incisional biopsies, except for three patients where the assay gave values below the 20% red colony threshold with one sample, but a mutant value with the other two samples. These results are most likely due to heterogeneity in the biopsies themselves, rather than any defect in the assay. This heterogeneity can take the form of either variation in tumour genotype or variation in tumour content in the biopsy. Eleven samples taken by trucut biopsy should have been excluded (RT–PCR failure in three cases, <20% tumour cells in eight cases). However in all these tumours the p53 status could be assessed using the second trucut. When we designed this study we were concerned with the fact that with trucut biopsies the physician is unable to confirm visually that actual tumour has been sampled. Even correct placement of the needle within the tumour mass does not guarantee that the sample will contain viable tumour cells, because tumours contain large areas of necrotic and fibrotic material. To address these concerns, we decided in this pilot study to take two trucut biopsies from each patient. This doubles the amount of work involved but the results obtained have vindicated this decision. Three samples taken by incisional biopsy gave less than 20% tumour cells and should have been excluded. Overall, if the 20% cut-off for tumour cell content and 20% cut-off for red colonies is applied, p53 status could be defined in 90% of patients by single incisional biopsy and 97% of patients by double trucut biopsy.

In conclusion we have shown in this study that p53 status can reliably be assigned by yeast assay using two trucut biopsies from the same tumour instead of a single incisional biopsy. Use of trucut biopsies will facilitate inclusion of patients in the EORTC 10994/BIG 00-01 clinical trial of neoadjuvant chemotherapy prior to surgery. This pilot study underlined the importance of performing histological examination before p53 testing in order to exclude cases where the p53 result may reflect only the status of the normal cells in the biopsy. In the EORTC 10994/BIG 00-01 trial a cryostat section of every biopsy will be examined histologically and only cases with 20% or more of tumour cells will be included in the study. To determine whether p53 status can more accurately be determined by chip techniques, a subset of samples from this study will also be tested on microarrays.
